# Antibacterial Effectiveness of Four Concentrations of the Hydroalcoholic Extract of *Solanum tuberosum* (*Tocosh*) against *Streptococcus mutans* ATCC 25175^TM^: A Comparative *In Vitro* Study

**DOI:** 10.1155/2020/8856382

**Published:** 2020-09-26

**Authors:** Silvana Enciso, Julia Medina, Franco Mauricio, Cesar Mauricio-Vilchez, Daniel Alvitez-Temoche, Luzmila Vilchez, Frank Mayta-Tovalino

**Affiliations:** ^1^Academic Department, Universidad Nacional Federico Villarreal, Lima, Peru; ^2^School of Dentistry, Universidad Nacional Mayor de San Marcos, Lima, Peru; ^3^Postgraduate Department, Universidad Cientifica del Sur, Lima, Peru

## Abstract

**Objective:**

To determine the *in vitro* antibacterial effect of four concentrations of the hydroalcoholic extract of *Solanum tuberosum* “*tocosh*” (HET) against *Streptococcus mutans* ATCC 25175^TM^.

**Methods:**

This was a prospective, experimental, comparative study. Fermented *tocosh* was subjected to hydric stress to obtain a hydroalcoholic extract at four different concentrations: 100%, 50%, 75%, and 25%. *S. mutans* strains were cultured in brain heart infusion agar using the swab technique. The antibacterial effectiveness of HET was evaluated following the Kirby–Bauer disk diffusion method and compared with 0.12% chlorhexidine (positive control group).

**Results:**

The highest mean inhibitory effect was achieved with HET at 100% (33.1 ± 2.2 mm, showing a gradual reduction in the other HET groups at 75%, 50%, and 25% (29.7 ± 1.3 mm, 26.6 ± 2.0, and 20.1 ± 1.8 mm, respectively)). Inferential analysis found statistically significant differences among all the experimental groups (*p*=0.001). The post hoc analysis also showed significant differences among all the experimental groups evaluated; however, there were no significant differences between HET 50% and chlorhexidine 0.12% (*p* > 0.05).

**Conclusions:**

It was found that the highest antibacterial effectiveness was obtained by HET 100%, being even higher than the 0.12% chlorhexidine positive control, and was statistically significant. Post hoc analysis showed that almost all the concentrations showed optimal efficacy against *S. mutans.*

## 1. Introduction

Throughout history, numerous investigations have shown that man has always used plants for medicinal purposes (preventive and curative), having numerous versatile applications [1]. The oral cavity is home to countless microorganisms in an ecosystem of considerable complexity. These microorganisms constitute the oral flora of humans which is highly diverse and can be easily altered by external factors, triggering manifestations leading to oral diseases [2–7].

In recent decades, certain protocols that help control bacterial flora have been established, including recommendations for improving oral hygiene, reducing the amount of carbohydrates and carbohydrates in the daily diet, oral physiotherapy programs that help improve mechanical removal of bacterial plaque (either individually or assisted), and also the use of substances based on natural products that contribute to the control of bacterial plaque in order to decrease the prevalence of tooth decay in the country. Even with all these improvement proposals, Peru continues to have an alarming prevalence of dental caries, which may be due to the difficult access that numerous regions of the country have to the prevention programs proposed so far [2–7].

In order to develop a product that may be used to prevent the appearance of dental caries in the high Andean population, knowledge of the biodiversity of different climates in Peru favoring the cultivation of natural products that can be used to solve this problem is necessary [2, 4]. In this regard, the use of *Solanum tuberosum* “*tocosh*,” a potato fermented through a process of water stress, that is native to and traditionally used in the Andean areas of Peru, may be a possible new therapeutic alternative. According to some studies, *tocosh* has important medicinal properties. However, there is still little evidence of its antibacterial effectiveness against bacteria associated with dental caries [2, 8].

Therefore, the aim of this study was to determine the *in vitro* antibacterial efficacy of four different concentrations of the hydroalcoholic extract of *tocosh* (HET) against *S. mutans* ATCC 25175.

## 2. Materials and Methods

### 2.1. Design and Sample Size

This experimental, prospective, comparative study was carried out in the Microbiology Laboratories of the Faculty of Natural Sciences of Universidad Nacional Federico Villarreal and the Laboratory of Plant Anatomy and Pharmacognosy of Universidad Nacional Mayor de San Marcos. With the help of a pilot study that provided the means and standard deviations, the sample size was calculated using the mean comparison formula (software Stata® 15), and an alpha value of 0.05 and a beta value of 0.8 were established, determining a sample size of *n* = 20 for each group.

### 2.2. Allocation

The following 6 groups were formed:

  Group 1: *S. mutans* ATCC 25175™ vs. 25% HET
  Group 2: *S. mutans* ATCC 25175™ vs. 50% HET
  Group 3: *S. mutans* ATCC 25175™ vs. 75% HET
  Group 4: *S. mutans* ATCC 25175™ vs. 100% HET
  Group 5: *S. mutans* ATCC 25175™ vs. chlorhexidine 0.12%
  Group 6: *S. mutans* ATCC 25175™ vs. distilled water

#### 2.2.1. Inclusion Criteria

- Petri dishes correctly inoculated with *S. mutans* ATCC 25175™ strains, each with the same amount of brain heart infusion (BHI) agar
- HET at 25%, 50%, 75%, and 100% prepared and packaged under sterile conditions

#### 2.2.2. Exclusion Criteria

- Petri dishes inoculated with *S. mutans* ATCC 25175 strains, presenting contamination and/or alterations due to poor incubation or poor operator manipulation
- HET at concentrations other than those required in the study

### 2.3. HET Preparation

*Tocosh* was collected from the city of Tarma, Peru, located in the Junín Department with the coordinates −11.4167°, −75.6833°. The sample provided was taken to the Laboratory of Plant Anatomy and Pharmacognosy and stored in a sterile glass container weighing 2 kg. The sample was handled directly since it is a fermented product. Then, the 60° hydroalcoholic solution was prepared from 96° ethyl alcohol and bidistilled water. The sample was placed into two 20 L glass containers, maintaining a ratio of 1 : 10 of the sample/volume of hydroalcoholic solution (1 kg of sample/10 L of solution), for a period of 10 days. Homogenization movements were periodically carried out daily. Subsequently, the solution was filtered and poured into 5 L beakers and placed in a water bath at a temperature of 40°C until the solvent evaporated or a soft mass was obtained. Finally, the extract was obtained by scraping and stored in amber glass containers. The entire procedure was carried out in the Plant Anatomy and Pharmacognosy Laboratory of the Faculty of Biological Sciences of the National University of San Marcos with voucher no. DI001-1218.

### 2.4. Obtaining and Reactivating the Strain

The strain used in the antimicrobial activity test corresponded to the American Type Culture Collection (ATCC), which is an international reference of *S. mutans* with the code ATCC® 25175^TM^ Lot 266-26-4 / 2020-02-290266 P from Laboratorio Gen Lab del Perú S.A.C. Before cultivation, the strain, which was stored at −80°C, was reactivated by a reactivation step 48 hours before the experiment and then remained preserved at 37°C.

### 2.5. Preparation of the Inoculum

The inoculum was prepared by means of the *S. mutans* colonies which were inoculated in BHI broth at 37°C. Turbidity density was standardized with McFarland turbidity scale 0.5, equivalent to a suspension of 1.5 × 10^8^ bacteria per ml.

### 2.6. *In Vitro* Antibacterial Test

A total of 20 wells for each group with plates of the BHI medium were prepared, each with 15 ml of the medium. Subsequently, selective seeding was performed using the swab technique on the BHI culture medium. A 6 mm diameter punch was used to prepare the wells with HET. Four equidistantly distributed wells in each culture plate contained 35 *µ*l of 25%, 50%, 75%, and 100% HET. 35 *μ*l of 0.12% chlorhexidine was used as the control and was immediately placed for bacterial control in culture media. Thereafter, the plates were incubated at 37°C, and reading was carried out after 24 hours using the Kirby–Bauer disk diffusion method. Antimicrobial efficacy was analyzed according to the formation of the inhibitory halo around the wells. The measurement was performed with a Mitutoyo Series 500 vernier ruler, which determined the diameter of the inhibition halo in mm.

### 2.7. Ethical Statement

Prior approval was requested for the execution of this study from the Office of Degrees and Titles of Universidad Nacional Federico Villarreal, and permission was also requested for the use of the Microbiology Laboratories of the Faculty of Natural Sciences of the same according to RR 3518-2006-UNFV.

### 2.8. Statistical Analysis

Descriptive analysis was performed by obtaining means and standard deviations as the main measures of central tendency. Furthermore, normal distribution of the quantitative variables was determined using the Shapiro–Wilk test. The homoscedasticity or homogeneity of variances was evaluated using the Bartlett test. The ANOVA test was used to perform the inferential analysis, and finally, the Bonferroni post hoc test was used to establish significant differences among each of the respective experimental groups. All the analyses were carried out establishing a level of significance of *p* < 0.05 and using Stata® 15 software.

## 3. Results

### 3.1. Evaluation of *In Vitro* Antimicrobial Activity

The highest mean inhibitory effect was obtained with HET at 100% (33.1 ± 2.2 mm), with a gradual reduction in the other HET groups at 75%, 50%, and 25% (29.7 ± 1.3 mm, 26.6 ± 2.0, and 20.1 ± 1.8 mm, respectively). All the groups presented a normal distribution with *p* > 0.05. Inferential analysis with the ANOVA test showed statistically significant differences among all the study groups (*p*=0.001) (Table 1).

### 3.2. Post Hoc Activity Test

Significant differences were found among all the experimental groups evaluated. However, no differences were observed between HET 50% and chlorhexidine 0.12% (*p* > 0.05) (Table 1).

## 4. Discussion

Currently, new natural product alternatives are being investigated to solve medical and dental problems. A new therapeutic alternative in our country is the use of the species *Solanum tuberosum* “*tocosh*,” a product native to and traditionally used in the Andean areas of Peru. Multiple studies have demonstrated its important medicinal properties. However, its antibacterial effectiveness against *S. mutans*, which has shown a relevant role in the pathogenesis of different oral diseases, has not yet been demonstrated [3–5]. Tooth decay is one of the most prevalent diseases in Peru, and it is associated with different etiological factors. One of these factors is poor oral health during the first years of life due to limited accessibility to health services or inadequate practices in the prevention of oral diseases [2, 6, 7]. Therefore, it is necessary to exploit the natural resources available and find future phytotherapeutic substances that can be used for therapeutic purposes [9–13].

According to the results of this study, all the concentrations evaluated (HET 25%, 50%, 75%, and 100%) showed remarkable antimicrobial effectiveness with inhibition halos exceeding 20 mm in all the groups as well as the positive control (chlorhexidine 0.12%). It should also be mentioned that distilled water (negative control) was excluded from the analyses because it did not show any effectiveness. According to Kim et al. [14], this activity can be attributed to different antimicrobial proteins (peptides) since they fulfill a fundamental role in the defense systems of all living organisms. On the contrary, these peptides are known to possess potent antimicrobial effectiveness against certain germs.

For example, Mohamed et al. [15] evaluated the antibacterial activity of 3 essential oils extracted from *Lantana camara, Corymbia citriodora*, and *Cupressus sempervirens* against isolates of *R. solanacearum*. Similarly, Mendieta et al. [16] evaluated aspartic proteases (StAP) from *Solanum tuberosum* and demonstrated that they are effective against *Fusarium solani* and *Phytophthora infestans*, with similar results to those described in our study.

The results of another study that also coincide with our findings is the study by Bártová et al. [17] who reported that the protease inhibitors of potato I and II reduced the growth of *P. infestans, Rhizoctonia solani*, and *Botrytis cinerea* and of the fungi of the genus *Fusarium*. These inhibitors were also able to inhibit certain microorganisms such as *Staphylococcus aureus, Listeria monocytogenes, Escherichia coli*, and *Candida albicans*. These authors therefore concluded that the proteins of *S. tuberosum* have optimal properties to inhibit the development of certain pathogens.

Furthermore, Ventrella et al. [9] evaluated the effects of potato and tomato extracts and their main components of *α*-solanine, *α*-chaconine, and *α*-tomatine glycoalkaloids on the development and reproduction of *Drosophila melanogaster*. They found that the biomodels exposed to the metabolites of the extracts increased certain alterations in the structure of the larvae, concluding that *Solanaceae* (nightshades) may be an important source of molecules that could be effectively used in agriculture. Our results were also similar to those of a previous study conducted by Mayta-Tovalino et al. [2] who reported that *tocosh* is a potato that has undergone a process of hydraulic oxidation that improves its antimicrobial properties and allows this natural resource to be used in medical sciences. To evaluate the antibacterial effect, the Kirby–Bauer halo inhibition method was used in *S. mutans* (ATCC 25175), *S. aureus* (ATCC 25923), and *Streptococcus mitis* (ATCC 49456), and it was concluded that this natural resource presented optimal antimicrobial activity against the tested oral strains.

The main limitations of this study were that it was only possible to evaluate the efficacy of *tocosh* against one of the main strains of the caries process. However, there are other microbial strains that also influence the development of certain oral diseases. Another limitation was that there is little evidence related to *tocosh*, although there are abundant studies regarding *S. tuberosum*. Nevertheless, it is important to remember that *tocosh* is a potato that has undergone constant water stress that potentially alters its conventional photochemical properties and characteristics. Another limitation of this research was that we did not use any concentration-based alcoholic solution as a control group. However, distilled water was used as a negative control since there were already too many experimental groups to carry out. Therefore, the relevance of the present study lies in that it is original, and there is a lack of studies on the antibacterial activity of *Solanum tuberosum* “*tocosh*” in *S. mutans*, and the results of this study provide a scientific basis for the use of *tocosh* as an alternative therapy in the control of oral diseases. This study is also important in the search for alternative products with proven antimicrobial action, which can be obtained from the flora of Peru. In addition, the results of this study have a high social impact due to the diversity of climates and a nature free of industries in the Andean region, allowing abundant harvesting of potatoes and promotion of the exploitation of fermented potatoes as an alternative preventive method which can be safely and routinely used in dental practice in high Andean populations.

Finally, *in vivo* studies are needed to assess the effectiveness and toxicity that the active components of *tocosh* can provide in order to verify the *in vitro* results. The effectiveness of HET against other bacteria that inhabit the oral cavity should also be evaluated, and comparative studies should be carried out using other types of extracts, such as ethanolic and methanolic, among others. It is necessary to seek synergism with other natural substances that can potentiate the antibacterial effect of this extract, and thus carry out research using this extract as an active ingredient for the preparation of antiseptic substances in the oral cavity.

## 5. Conclusions

According to the results obtained and within the limitations of this *in vitro* investigation, it was shown that HET at 100% and 75% had the highest inhibition halo, being even greater than the 0.12% chlorhexidine positive control, and the result was statistically significant. Post hoc analysis showed no differences between the inhibitory halos of HET 50% and chlorhexidine 0.12%, and HET 25% presented a lower inhibition halo compared to chlorhexidine. Nonetheless, the majority of concentrations showed optimal efficacy against *S. mutans.*

## Figures and Tables

**Table 1:** Inhibition halo of HET against the *S. mutans* strain.

Groups	Mean	SD	Min	Max	*p* ^*∗*^	*p* ^*∗∗*^
HET 25%	20.2^d^	1.8	17.0	24.0	>0.050	0.001
HET 50%	26.6^c^	2.0	23.0	30.0		
HET 75%	29.7^b^	1.3	28.0	32.0		
HET 100%	33.1^a^	2.2	30.0	36.0		
Chlorhexidine 0.12%	26.0^c^	2.5	21.0	30.0		

Inhibition halos were measured in mm. The DW negative control was excluded from the statistical analyses because it did not present antimicrobial efficacy against *S. mutans*. HET = hydroalcoholic extract of *tocosh*, CHx = chlorhexidine, and DW = distilled water. ^*∗*^Shapiro–Wilk test. ^*∗∗*^ANOVA test. ^a,b,d^Bonferroni post hoc test: post hoc test of the antibacterial effectiveness of HET against *S. mutans* was statistically significant in all groups (*p* < 0.001). ^c^However, no differences were observed between HET 50% and chlorhexidine 0.12% (*p* > 0.05).

## Data Availability

The data supporting the results of this study are available after authorization and request from the corresponding institution.
